# Supplementary material for: Gene polymorphism associated with angiotensinogen (M235T), endothelial lipase (584C/T) and susceptibility to coronary artery disease: a meta-analysis
Source: Biosci Rep. 2020 Jul 24;40(7):BSR20201414. doi: 10.1042/BSR20201414 (PMC7383830; doi:10.1042/BSR20201414)
Supplement: Supplementary Table S1 [file BSR-2020-1414_supp.pdf]

Supplementary table 1 Quality assessment included in the study(NOS)

		Case selection				Comparability between groups		Exposure factor measurement			
First author	Year	1	2	3	4	5	6	Blind method	7	Response rate	NOS score
Ko YL	1997	*	*	*	*	*	*	?	*	*	8
Chen D	1998	*	*	*	*	*	*	?	*	*	8
Sheu WH	1998	*	*	*	*	*	*	?	*	?	7
Xie Y	2001	*	*	*	*	*	*	?	*	?	7
Zhu TB	2002	*	*	*	*	*	*	?	*	*	8
Gu WD	2003	*	*	*	*	*	*	?	*	*	8
Zhu TN	2004	*	*	*	*	*	*	?	*	*	8
Li L	2004	*	*	*	*	*	*	?	*	?	7
Ren J	2005	*	*	*	*	*	*	?	*	?	7
Yang JP	2013	*	*	*	*	*	*	?	*	?	7
Huang YZ	2013	*	*	*	*	*	*	?	*	?	7
Al-Hazzani	2014	*	*	*	*	*	*	?	*	*	8
Bonfim a	2016	*	*	*	*	*	*	?	*	*	8
Bonfim b	2016	*	*	*	*	*	*	?	*	*	8
Khatami	2017	*	*	*	*	*	*	?	*	*	8
Isordia-Salas	2018	*	*	*	*	*	*	?	*	*	8
Zhu M	2019	*	*	*	*	*	*	?	*	*	8
Rimm	1992	*	*	*	*	*	*	?	*	*	8
Colditz	1997	*	*	*	*	*	*	?	*	*	8
Tjonneland	2007	*	*	*	*	*	*	?	*	*	8
Zhu JC	2007	*	*	*	*	*	*	?	*	?	7
Tang NP	2008	*	*	*	*	*	*	?	*	*	8
Zhang L	2009	*	*	*	*	*	*	?	*	?	7
Cai GJ	2014	*	*	*	*	*	*	?	*	*	8
Xie L	2015	*	*	*	*	*	*	?	*	*	8
Elnaggar	2018	*	*	*	*	*	*	?	*	*	8
Solim	2018	*	*	*	*	*	*	?	*	*	8
Shimizu	2007	*	*	*	*	*	*	?	*	*	8
Vergeer	2010	*	*	*	*	*	*	?	*	*	8
Toosi	2015	*	*	*	*	*	*	?	*	*	8

Note:*:Yes;?:unclear;1:Case identification appropriate;2:Case representativeness;3:The source of the control clear;4:control group choosed properly;5:Controls the most important confounding factors;6:Control other confounding factors;7:same exposure determination method; NOS:Newcastle-Ottawa Scale
